# Supplementary figures and images for: The Performance Analysis of PSO-ResNet for the Fault Diagnosis of Vibration Signals Based on the Pipeline Robot
Source: Sensors (Basel). 2023 Apr 26;23(9):4289. doi: 10.3390/s23094289 (PMC10181494; doi:10.3390/s23094289)

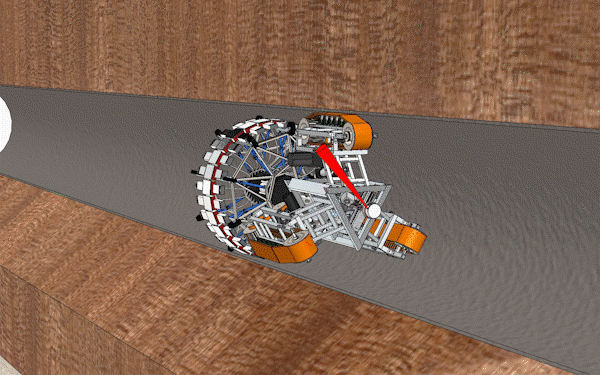

Supplement: Supplementary file 1 [file sensors-23-04289-s001.zip › Figure S1. Illustration of pipeline robot operation status.gif]

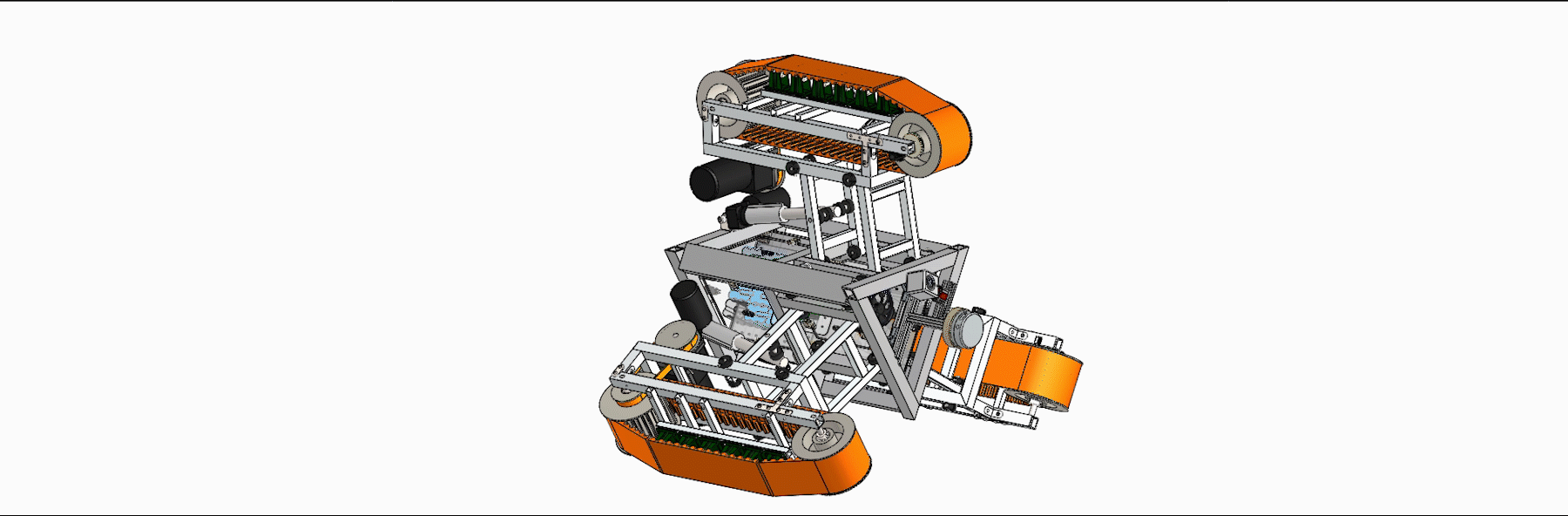

Supplement: Supplementary file 1 [file sensors-23-04289-s001.zip › Figure S2. Schematic diagram of pipeline robot structure.GIF]
